# Novel Decomposition Technique on Rational-Based Neuro-Transfer Function for Modeling of Microwave Components

**DOI:** 10.3390/mi11070696

**Published:** 2020-07-17

**Authors:** Zhihao Zhao, Feng Feng, Jianan Zhang, Wei Zhang, Jing Jin, Jianguo Ma, Qi-Jun Zhang

**Affiliations:** 1 School of Microelectronics, Tianjin University, Tianjin 300072, China; zhihaozhao@doe.carleton.ca (Z.Z.); jingjin5@cmail.carleton.ca (J.J.); qjz@doe.carleton.ca (Q.-J.Z.); 2 Department of Electronics, Carleton University, Ottawa, ON K1S5B6, Canada; jiananzhang@doe.carleton.ca (J.Z.); weizhang13@doe.carleton.ca (W.Z.); 3 School of Computer Science and Technology, Guangdong University of Technology, Guangzhou 510006, China; mjg@gdut.edu.cn

**Keywords:** decomposition, microwave components, neural networks, parameter extraction, parametric modeling, rational-based transfer function

## Abstract

The rational-based neuro-transfer function (neuro-TF) method is a popular method for parametric modeling of electromagnetic (EM) behavior of microwave components. However, when the order in the neuro-TF becomes high, the sensitivities of the model response with respect to the coefficients of the transfer function become high. Due to this high-sensitivity issue, small training errors in the coefficients of the transfer function will result in large errors in the model output, leading to the difficulty in training of the neuro-TF model. This paper proposes a new decomposition technique to address this high-sensitivity issue. In the proposed technique, we decompose the original neuro-TF model with high order of transfer function into multiple sub-neuro-TF models with much lower order of transfer function. We then reformulate the overall model as the combination of the sub-neuro-TF models. New formulations are derived to determine the number of sub-models and the order of transfer function for each sub-model. Using the proposed decomposition technique, we can decrease the sensitivities of the overall model response with respect to the coefficients of the transfer function in each sub-model. Therefore, the modeling approach using the proposed decomposition technique can increase the modeling accuracy. Two EM parametric modeling examples are used to demonstrate the proposed decomposition technique.

## 1. Introduction

Parametric modeling of electromagnetic (EM) behaviors for microwave components has become important for EM-based design in the microwave area. EM-based designs can be time-consuming, since repetitive simulations of EM are usually required due to value adjustments of geometrical parameters. Parametric models are developed to characterize the relationship between geometrical variables and EM responses. The developed parametric models allow faster simulation and optimization with varying values of geometrical parameters and can be subsequently implemented in high-level design of circuit and system.

An artificial neural network (ANN) has been an important vehicle for parametric modeling of EM behavior in radio frequency and microwave area [1,2,3,4,5,6]. ANN benefits from its strong learning and generalization capabilities and it has been used for a wide variety of microwave applications [7,8,9,10,11,12,13,14,15,16]. The universal approximation theorem [17] of ANNs provides the theoretical foundation that if sufficient data are used in training, the good accuracy of ANN models can be achieved within the training region. To develop an accurate parametric model, we train ANNs to learn the nonlinear relationship between geometrical variables and EM behavior. Apart from modeling microwave passive components, ANN has also been used for modeling microwave active components, such as power amplifiers [18], field-effect transistor [19], high electron-mobility transistors [20], etc.

Another popular parametric modeling method is the neuro-transfer function (neuro-TF) method [21,22,23,24,25], which combines neural networks and transfer functions. In the neuro-TF method, EM responses of passive components versus frequency are represented by the transfer functions. The transfer functions in the neuro-TF model are used as prior knowledge, which allows less hidden neurons and less training data to be used in the model development than the ANN method. The prior knowledge can help speed up model development and enhance the capability for learning and generalization of the overall model. One of the popular neuro-TF methods is the pole-residue-based neuro-TF method [22,23]. This method can deal with high-order problem. However, when the geometrical variations become large, some poles and residues obtained from vector fitting may vary discontinuously with respect to geometrical variables. This discontinuity issue leads to the difficulty of training for the overall model. Hybrid-based neuro-TF method [24] is used to further reduce the discontinuity issue of poles and residues by converting the most discontinuous poles and residues into the rational format. Rational-based neuro-TF method [25] is an alternative neuro-TF method, which does not have the discontinuity issue of poles and residues. The rational-based neuro-TF works well when the order in the neuro-TF model is low. However, as the order increases, the transfer function response is very sensitive to the coefficients of the transfer function, leading to the high-sensitivity issue. The high-sensitivity issue can result in the difficulty of training for the overall model when the order in the neuro-TF model is high. How to solve the high-sensitivity issue in the rational-based neuro-TF modeling method is a challenging topic.

This paper proposes a new decomposition technique for developing a rational-based neuro-TF model for EM microwave components, addressing the challenge of high-sensitivity issue. The proposed technique decomposes the single neuro-TF model with high order into multiple sub-neuro-TF models with much lower order and reformulates the overall neuro-TF model as the combination of the sub-neuro-TF models. New formulations have been derived for determination of the number of sub-neuro-TF models and the order of each sub-neuro-TF model. The proposed decomposition technique can decrease the sensitivities of the overall model response with respect to the coefficients of the transfer function, improving the overall model accuracy over the existing rational-based neuro-TF method. Compared with the existing pole-residue-based neuro-TF method, the proposed neuro-TF method has fewer sub-neuro-TF models and less discontinuity issue in each sub-neuro-TF model, achieving better overall model accuracy.

We organize this paper as follows. Section 2 describes the high-sensitivity issue in the existing rational-based neuro-TF method. Section 3 provides a detailed description of the proposed decomposition technique and the proposed decomposition technique for developing the proposed rational-based neuro-TF model. Section 4 demonstrates the proposed decomposition technique by two EM examples. Section 5 concludes the paper.

## 2. The High-Sensitivity Issue in the Existing Rational-Based Neuro-TF Method

The existing rational-based neuro-TF modeling method [25] requires a parameter extraction process for extraction of the coefficients of the transfer function numerator and denominator in the neuro-TF model for each geometrical parameter sample. The existing method can be well-functioning if the geometrical variations are small and/or the order of the transfer function in the neuro-TF model is low. However, the existing neuro-TF method may not be suitable to address the situations where geometrical variations become large and/or where the order of the transfer function becomes high.

When the geometrical variations are small, the relationship between the geometrical variables and the coefficients of the transfer function is of low nonlinearity. Since neural networks can easily learn this relationship, the training accuracy of the neural networks is high. This results in the high training accuracy of the overall neuro-TF model. When the geometrical variations become larger, the relationship between the geometrical variables and the coefficients of the transfer function becomes more nonlinear. The accuracy of the neural networks becomes lower. Under this situation, the order of the neuro-TF model becomes an important factor in the accuracy of the overall model. When the transfer function order becomes high, the sensitivities of the neuro-TF model response with respect to the coefficients of the transfer function become high. With the high sensitivity, a relatively small error of the neural networks will result in a large error of the overall neuro-TF model. How to solve this high-sensitivity issue to increase the accuracy of the rational-based neuro-TF is a challenging topic.

To address the high-sensitivity issue, this paper proposes a novel decomposition technique for the rational-based neuro-TF modeling method. The main idea is that we decompose the neuro-TF model into sub-neuro-TF models and reformulate the overall neuro-TF model as the combination of the sub-neuro-TF models. In this way, the order of the overall neuro-TF model is decomposed into lower orders of the sub-neuro-TF models, decreasing the sensitivities of the overall model response with respect to the coefficients of the transfer function. By decreasing sensitivities of the overall model response with respect to coefficients of the transfer function using the proposed decomposition technique, the accuracy of the overall neuro-TF model can be improved.

## 3. Proposed Decomposition Technique for Development of Rational-Based Neuro-TF Model

### 3.1. Concept of the Decomposition Technique for Rational-Based Neuro-TF Model

Let x be a vector which contains the geometrical variables. Let *y* represent the frequency response, e.g., *S*-parameter. A general rational-based neuro-TF model [25] in frequency domain can be expressed as

(1) $$y\left(x,w_{a},w_{b},s\right)=\frac{\sum_{j=1}^{N}a_{j}\left(x,w_{a}\right)s^{j-1}}{1+\sum_{j=1}^{N}b_{j}\left(x,w_{b}\right)s^{j}}$$

where *N* represents the order of the transfer function; $a_{j}$ and $b_{j}$ represent the *j*th coefficients of the transfer function numerator and denominator, respectively; $w_{a}$ and $w_{b}$ represent the neural network weights; and *s* represents the Laplace domain frequency.

As can be seen from (1), the direct decomposition of the rational-based neuro-TF is not easy. In this case, we propose to reformat the rational-based transfer function and decompose the neuro-TF model indirectly. To reformat the rational-based transfer function, we use the pole-residue-based transfer function [23], which is another format of the transfer function. Here we define the effective poles as the poles whose imaginary parts are positive [23]. We define the effective residues as the residues corresponding to the effective poles [23]. We define the effective order as the order of the pole-residue transfer function which consists only of the effective poles and residues. In order to derive the decomposition technique for the rational-based neuro-TF model, we need to first convert the rational-based transfer function into the pole-residue format, expressed as

(2) $$y=\frac{\sum_{j=1}^{N}a_{j}s^{j-1}}{1+\sum_{j=1}^{N}b_{j}s^{j}}=\sum_{j=1}^{N_{eff}}\frac{r_{j}}{s-p_{j}}+\sum_{j=1}^{N_{eff}}\frac{r_{j}^{*}}{s-p_{j}^{*}}$$

where $N_{eff}$ is the effective order of the pole-residue-based transfer function; $p_{j}$ and $r_{j}$ are the *j*th effective pole and residue of the pole-residue-based transfer function, respectively; and $p_{j}^{*}$ and $r_{j}^{*}$ are the complex conjugate parts of *j*th effective pole and residue, respectively. To ensure that all the poles and residues in (2) are in complex values, order *N* should be an even number and thus the effective order $N_{eff}=N/2$. It is noticed that the pole-residue-based transfer function itself is expressed as a summation of sub-transfer functions. We can easily decompose the pole-residue-based transfer function into multiple sub-transfer functions in pole-residue format. Let *M* represent the number of the sub-transfer functions. The decomposition of the pole-residue-based transfer function can be expressed as

(3) $$y=\sum_{i=1}^{M}y_{i}=\sum_{i=1}^{M}\left(\sum_{j=1}^{N_{i}}\frac{r_{n(i,j)}}{s-p_{n(i,j)}}+\sum_{j=1}^{N_{i}}\frac{r_{n(i,j)}^{*}}{s-p_{n(i,j)}^{*}}\right)$$

where

(4) $$n\left(i,j\right)=\left\{\begin{array}{c} j,\;\;\;\;\;\;\;\;\;\;\;\;\;\;\;\;\;\;\;\;\;\;\;\;\;\;\;\;\;\;\;\mathrm{if}\;\;i=1\\ j+\sum_{t=1}^{i-1}N_{t},\;\;\;\;\;\;\;\;\;\;\;\;\;\;\;\mathrm{if}\;\;i>1 \end{array},\right.$$

$y_{i}$ represents the frequency response of the *i*th sub-transfer function; and $N_{i}$ represents the effective order of the *i*th sub-transfer function. The relationship between $N_{eff}$, $N_{i}$, and *M* can be expressed as

(5) $$N_{eff}=\sum_{i=1}^{M}N_{i}.$$

After the decomposition, we convert these sub-transfer functions back into the rational format, expressed as

(6) $$y_{i}=\sum_{j=1}^{N_{i}}\frac{r_{n(i,j)}}{s-p_{n(i,j)}}+\sum_{j=1}^{N_{i}}\frac{r_{n(i,j)}^{*}}{s-p_{n(i,j)}^{*}}=\frac{\sum_{j=1}^{2N_{i}}{\hat{a}}_{ij}s^{j-1}}{1+\sum_{j=1}^{2N_{i}}{\hat{b}}_{ij}s^{j}}$$

where ${\hat{a}}_{ij}$ and ${\hat{b}}_{ij}$ represent the *j*th coefficients of the numerator and denominator for the *i*th sub-transfer function, respectively. Now the rational-based transfer function can be formulated as the summation of *M* sub-rational-based transfer functions. Substituting (6) into (3), we derive the rational-based neuro-TF model incorporating decomposition technique, formulated as

(7) $$y\left(x,w_{a},w_{b},s\right)=\sum_{i=1}^{M}y_{i}\left(x,w_{a},w_{b},s\right)=\sum_{i=1}^{M}\frac{\sum_{j=1}^{2N_{i}}{\hat{a}}_{ij}\left(x,w_{a}\right)s^{j-1}}{1+\sum_{j=1}^{2N_{i}}{\hat{b}}_{ij}\left(x,w_{b}\right)s^{j}}.$$

Table 1 illustrates the sensitivities of the proposed and the existing neuro-TF model responses with respect to coefficients of the transfer function. In Table 1, for the existing neuro-TF model formulated in (2), when the frequency range is small (e.g., varying around 1 to ignore the influence of $s^{j}$ or $s^{j-1}$), all the effective poles (i.e., $p_{j},\forall j\in N$) are close to each other. When the value of frequency *s* is in the middle of all the effective poles, the value of $\prod_{j=1}^{N_{eff}}\left(s-p_{j}\right)$ becomes very small as $N_{eff}$ is high, which makes the *y* too sensitive with respect to the coefficients $a_{j}$ and $b_{j}$. This high sensitivity-issue cannot be easily solved by simply reducing the order *N* in vector fitting, since the minimum value of *N* has been used in vector fitting. The high-sensitivity issue can be effectively overcome by the neuro-TF model incorporating the proposed decomposition technique, formulated in (7). For the proposed neuro-TF model, the value of $N_{i}$ is much smaller than that of $N_{eff}$. In that case, the value of $\prod_{j=1}^{N_{i}}\left(s-p_{n(i,j)}\right)$ is much larger than that of $\prod_{j=1}^{N_{eff}}\left(s-p_{j}\right)$. Therefore, the sensitivities of *y* with respect to the coefficients ${\hat{a}}_{ij}$ and ${\hat{b}}_{ij}$ of the proposed neuro-TF model are much lower than those with respect to the coefficients $a_{j}$ and $b_{j}$ of the existing neuro-TF model. With lower sensitivities of the model response with respect to its coefficients of the transfer function, the proposed neuro-TF model can achieve higher accuracy than the existing neuro-TF model.

We use ${\hat{a}}_{i}$ and ${\hat{b}}_{i}$ to represent vectors which contain all the coefficients of the transfer function numerator and denominator in the *i*th sub-neuro-TF model, respectively, defined as

(8) $${\hat{a}}_{i}={\left[{\hat{a}}_{i1}\;{\hat{a}}_{i2}\;\cdots \;{\hat{a}}_{ij}\;\cdots \;{\hat{a}}_{i(2N_{i})}\right]}^{T}$$

and

(9) $${\hat{b}}_{i}={\left[{\hat{b}}_{i1}\;{\hat{b}}_{i2}\;\cdots \;{\hat{b}}_{ij}\;\cdots \;{\hat{b}}_{i(2N_{i})}\right]}^{T}$$

where i=1,2,...,M and $j=1,2,...,2N_{i}$. Based on the definition of coefficients ${\hat{a}}_{i}$ and ${\hat{b}}_{i}$, we use $\hat{a}$ and $\hat{b}$ to represent vectors which contain all the coefficients of the transfer function numerators and denominators in all the sub-neuro-TF models, respectively, defined as

(10) $$\hat{a}={\left[\;{\hat{a}}_{1}^{T}\;\;{\hat{a}}_{2}^{T}\;\cdots \;{\hat{a}}_{i}^{T}\;\cdots \;{\hat{a}}_{M}^{T}\;\right]}^{T}$$

and

(11) $$\hat{b}={\left[\;{\hat{b}}_{1}^{T}\;\;{\hat{b}}_{2}^{T}\;\cdots \;{\hat{b}}_{i}^{T}\;\cdots \;{\hat{b}}_{M}^{T}\;\right]}^{T}$$

where i=1,2,...,M.

**Figure 1:**
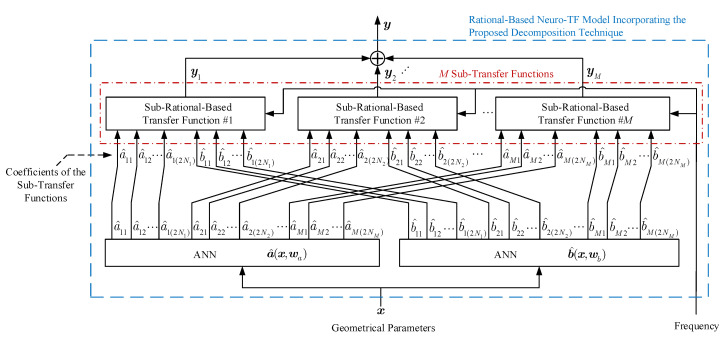
Structure for the rational-based neuro-TF model incorporating the proposed decomposition technique.

Figure 1 shows the structure of the neuro-TF model incorporating the proposed decomposition technique.

As can be seen in Figure 1, the proposed model consists of two separate neural networks. Neural network $\hat{a}\left(x,w_{a}\right)$ is used to learn and represent the nonlinear relationship between the geometrical variables x and the coefficients $\hat{a}$ of the transfer function numerators in all the sub-neuro-TF models. Neural network $\hat{b}\left(x,w_{b}\right)$ is used to learn and represent the nonlinear relationship between the geometrical variables x and the coefficients $\hat{b}$ of the transfer function denominators in all the sub-neuro-TF models.

In the next subsection, we propose a novel decomposition technique for the parameter extraction and development of the proposed rational-based neuro-TF model. New formulations are derived to determine the number *M* of sub-neuro-TF models and the effective order $N_{i}$ of the transfer function for each sub-neuro-TF model. Using the proposed decomposition technique, we can decrease the sensitivities of the overall model response with respect to the coefficients of the transfer function in each sub-model, achieving better overall model accuracy.

### 3.2. Proposed Decomposition Technique for Parameter Extraction and Model Development

In this subsection, we propose a decomposition technique for parameter extraction and model development. The proposed decomposition technique starts with the EM data samples, e.g., *S*-parameter data for different training samples of the geometrical parameter. Let $n_{s}$ represent the total number of training samples. Let $T_{r}$ denote the index set of the training samples of the geometrical variables, i.e., $T_{r}=\left\{1,2,...,n_{s}\right\}$. Let $x_{k}$ and $d_{k}$ represent the *k*th sample of the geometrical variables and EM data, respectively, where $k\in T_{r}$. During data generation, frequency is swept by the EM simulator as a separate variable.

We perform the vector fitting process [26] to obtain poles and residues for each geometrical parameter sample. A scaling-and-shifting process for the frequency range with an even number of order *N* is set up during the vector fitting process to get all the poles and residues in complex values. Let ${\tilde{p}}^{\left(k\right)}$ and ${\tilde{r}}^{\left(k\right)}$ represent vectors which contain the poles and the residues obtained after vector fitting, respectively, at the *k*th geometrical sample, defined as

(12) $${\tilde{p}}^{\left(k\right)}={\left[{\tilde{p}}_{1}^{\left(k\right)}\;{\tilde{p}}_{2}^{\left(k\right)}\;\cdots \;{\tilde{p}}_{\tilde{\ell }}^{\left(k\right)}\;\cdots \;{\tilde{p}}_{N}^{\left(k\right)}\right]}^{T}$$

and

(13) $${\tilde{r}}^{\left(k\right)}={\left[{\tilde{r}}_{1}^{\left(k\right)}\;{\tilde{r}}_{2}^{\left(k\right)}\;\cdots \;{\tilde{r}}_{\tilde{\ell }}^{\left(k\right)}\;\cdots \;{\tilde{r}}_{N}^{\left(k\right)}\right]}^{T}$$

where ${\tilde{p}}_{\tilde{\ell }}^{\left(k\right)}$ and ${\tilde{r}}_{\tilde{\ell }}^{\left(k\right)}$ denote the $\tilde{\ell }$th related pole and residue, respectively; and $\tilde{\ell }\in \tilde{I}=\left\{1,2,...,N\right\}$. The obtained poles/residues contain both the effective poles/residues (i.e., the poles whose imaginary parts are positive/the residues that related to the effective poles) and the complex conjugate parts of the effective poles/residues.

In the first stage of the proposed decomposition technique, we sort the effective poles and residues in ascending sequence according to the values of the imaginary parts of the effective poles. The effective poles obtained from vector fitting may not be in the same sequence from sample to sample, which will result in the discontinuity of the effective poles over the geometrical parameters from sample to sample. The sorting process can make the effective poles located in the same sequence among different geometrical samples, minimizing the discontinuity of the effective poles. With minimum discontinuity issue in the effective poles, the proposed decomposition technique can be more robust, and the obtained sub-neuro-TF models can achieve better accuracy. Let the effective poles and residues after sorting be denoted as $p^{\left(k\right)}$ and $r^{\left(k\right)}$, respectively. The *n*th elements of $p^{\left(k\right)}$ and $r^{\left(k\right)}$ are denoted as $p_{n}^{\left(k\right)}$ and $r_{n}^{\left(k\right)}$, respectively, calculated as

(14) $$p_{n}^{\left(k\right)}={\tilde{p}}_{\ell_{n}}^{\left(k\right)}$$

and

(15) $$r_{n}^{\left(k\right)}={\tilde{r}}_{\ell_{n}}^{\left(k\right)}$$

where

(16) ℓn=argminℓ˜∈D\DnIm(p˜ℓ˜(k))

(17) D=ℓ˜Im(p˜ℓ˜(k))>0,ℓ˜∈I˜

(18) $$D_{n}=\left\{\begin{array}{c} Ø,\;\;\;\;\;\;\;\;\;\;\;\;\;\;\;\;\;\;\;\;\;\;\;\;\;\;\;\mathrm{if}\;\;n=1\\ D_{n-1}⋃\left\{\left.\ell_{n-1}\right\}\right.\;\;\;\;\;\;\;\;\;\;\;\mathrm{if}\;\;n>1 \end{array}\right.$$

and $n\in I=\left\{1,2,...,N_{eff}\right\}$, where $N_{eff}=N/2$.

The second stage of the proposed decomposition technique is to quantify the degree of smoothness of the sorted effective pole/residue data with respect to the changing of the geometrical variables. The sorted effective poles and residues are correlated with the values of geometrical variables when the values of geometrical variables vary continuously. The quantified degree of smoothness can be used as an indicator for decomposition. Let $\sigma_{n}^{p}$ represent the deviation of the *n*th effective pole as the geometrical parameter changes. Similarly, let $\sigma_{n}^{r}$ represent the deviation of the *n*th effective residue as the geometrical parameter changes. The deviations $\sigma_{n}^{p}$ and $\sigma_{n}^{r}$ are formulated as

(19) σnp=maxk∈TrRe(pn(k))−Re(μnp)Re(μnp)2+Im(pn(k))−Im(μnp)Im(μnp)21/2

and

(20) σnr=maxk∈TrRe(rn(k))−Re(μnr)Re(μnr)2+Im(rn(k))−Im(μnr)Im(μnr)21/2

where $\mu_{n}^{p}$ and $\mu_{n}^{r}$ are expressed as

(21) $$\mu_{n}^{p}=\frac{1}{n_{s}}\sum_{k=1}^{n_{s}}p_{n}^{\left(k\right)}$$

and

(22) $$\mu_{n}^{r}=\frac{1}{n_{s}}\sum_{k=1}^{n_{s}}r_{n}^{\left(k\right)}.$$

The deviations $\sigma_{n}^{p}$ and $\sigma_{n}^{r}$ are computed using all the geometrical samples to represent the variations of each sorted effective pole and residue. Here we define the deviation vector σ as

(23) $$\sigma ={\left[\sigma_{1},\sigma_{2},...,\sigma_{n},...,\sigma_{N_{eff}}\right]}^{T}$$

where

(24) $$\sigma_{n}=\sqrt{{\left(\sigma_{n}^{p}\right)}^{2}+{\left(\sigma_{n}^{r}\right)}^{2}}$$

and n∈I. The deviation vector σ reflects the degree of smoothness of the effective pole/residue data ($p^{\left(k\right)}$ and $r^{\left(k\right)}$). It can be seen that the size of σ is consistent with the size of the index set $I=\{1,2,...,N_{eff}\}$. Based on the different values of $\sigma_{n}$, the index set *I* will be decomposed into multiple subsets for parameter extraction.

In the third stage, we decompose the index set $I=\{1,2,...,N_{eff}\}$ into *M* subsets based on σ and *M*. The initial value of *M* is set to two. The ultimate value of *M* will be determined through an iterative decomposition process for the proposed rational-based neuro-TF model, which will be explained later on. Since the size of the *i*th subset depends on the effective order $N_{i}$ of the *i*th sub-neuro-TF model, we determine the effective order $N_{i}$, formulated as

(25) $$N_{i}=\left\{\begin{array}{c} \left\lceil \frac{N_{eff}}{M}\right\rceil ,\;\;\;\;\;\;\;\;\;\;\;\;\;\;\;\;\;\;\;\;\;\mathrm{if}\;\;i=1\\ \left\lceil \frac{N_{eff}-\sum_{j=1}^{i-1}N_{j}}{M-i+1}\right\rceil ,\;\;\;\;\mathrm{if}\;\;i>1 \end{array}\right.$$

where i=1,2,...,M. By using (25), $N_{i}$ is distributed as evenly as possible for each sub-neuro-TF model.

After the determination of $N_{i}$, we sort the elements of the deviation vector σ in descending sequence. Let $\hat{u}$ denote a vector which contains the indices of the elements of σ after sorting. Let ${\hat{u}}_{m}$ denote the *m*th element of $\hat{u}$, formulated as

(26) $${\hat{u}}_{m}=\arg\;\max_{u\in I\backslash J_{m}}\left\{\left.\sigma_{u}\right\}\right.$$

where

(27) $$J_{m}=\left\{\begin{array}{c} Ø,\;\;\;\;\;\;\;\;\;\;\;\;\;\;\;\;\;\;\;\;\;\;\;\;\;\;\;\;\;\mathrm{if}\;\;m=1\\ J_{m-1}⋃\left\{\left.{\hat{u}}_{m-1}\right\}\right.,\;\;\;\;\;\;\;\;\mathrm{if}\;\;m>1 \end{array}\right.$$

and m∈I. We define the *i*th subset $I_{i}\subseteq I$, where i=1,2,...,M; $\left|I_{i}\right|=N_{i}$; ${⋃}_{i=1}^{M}I_{i}=I$; and $\left|{⋃}_{i=1}^{M}I_{i}\right|=\left|I\right|=N_{eff}$. Using the information of the index vector u and the effective order $N_{i}$, the *i*th subset $I_{i}$ can be expressed as

(28) $$I_{i}=\left\{\left.{\hat{u}}_{q+1},{\hat{u}}_{q+2},...,{\hat{u}}_{q+N_{i}}\right\}\right.$$

where *q* can be formulated as

(29) $$q=\left\{\begin{array}{c} 0,\;\;\;\;\;\;\;\;\;\;\;\;\;\;\;\;\mathrm{if}\;\;i=1\\ \sum_{t=1}^{i-1}N_{t},\;\;\;\;\;\;\;\mathrm{if}\;\;i>1 \end{array}.\right.$$

In the fourth stage, we group the effective poles $p^{\left(k\right)}$ and their complex conjugate parts ${\left(p^{*}\right)}^{\left(k\right)}$ into pole subsets and group the effective residues $r^{\left(k\right)}$ and their complex conjugate parts ${\left(r^{*}\right)}^{\left(k\right)}$ into residue subsets, based on the information of subsets $I_{i},\forall i=1,2,...,M$. Let ${\hat{p}}_{i}^{\left(k\right)}$ and ${\hat{r}}_{i}^{\left(k\right)}$ denote the pole and residue data for the *i*th sub-neuro-TF model, respectively, expressed as

(30) $${\hat{p}}_{i}^{\left(k\right)}=\left[p_{{\hat{u}}_{q+1}}^{\left(k\right)}\;\;{\left(p_{{\hat{u}}_{q+1}}^{*}\right)}^{\left(k\right)}\;\;p_{{\hat{u}}_{q+2}}^{\left(k\right)}\;\;{\left(p_{{\hat{u}}_{q+2}}^{*}\right)}^{\left(k\right)}\right.{\left....\;\;p_{{\hat{u}}_{q+N_{i}}}^{\left(k\right)}\;\;{\left(p_{{\hat{u}}_{q+N_{i}}}^{*}\right)}^{\left(k\right)}\right]}_{1\times 2N_{i}}^{T}$$

and

(31) $${\hat{r}}_{i}^{\left(k\right)}=\left[r_{{\hat{u}}_{q+1}}^{\left(k\right)}\;\;{\left(r_{{\hat{u}}_{q+1}}^{*}\right)}^{\left(k\right)}\;\;r_{{\hat{u}}_{q+2}}^{\left(k\right)}\;\;{\left(r_{{\hat{u}}_{q+2}}^{*}\right)}^{\left(k\right)}\right.{\left....\;\;r_{{\hat{u}}_{q+N_{i}}}^{\left(k\right)}\;\;{\left(r_{{\hat{u}}_{q+N_{i}}}^{*}\right)}^{\left(k\right)}\right]}_{1\times 2N_{i}}^{T}$$

where i=1,2,...,M; and *q* is calculated by (29).

We convert these grouped pole and residue data (i.e., ${\hat{p}}_{i}^{\left(k\right)}$ and ${\hat{r}}_{i}^{\left(k\right)}$) into the coefficient data of the transfer function numerator ${\hat{a}}_{i}^{\left(k\right)}$ and the transfer function denominator ${\hat{b}}_{i}^{\left(k\right)}$ for the *i*th sub-neuro-TF model. Since the nonlinear relationships between the geometrical variables x and the coefficients of the numerators ${\hat{a}}_{i}$ are usually similar among different sub-neuro-TF models, we use one single neural network $\hat{a}\left(x,w_{a}\right)$ to learn the relationships between the geometrical variables x and the coefficients of the numerators $\hat{a}$ of all the sub-neuro-TF models [25]. We use another single neural network $\hat{b}\left(x,w_{b}\right)$ to learn the relationships between the geometrical variables x and the coefficients of the denominators $\hat{b}$ of all the sub-neuro-TF models. Let $\left(x_{k},{\hat{a}}^{\left(k\right)}\right)$ and $\left(x_{k},{\hat{b}}^{\left(k\right)}\right)$ denote the training data which are used to train the two neural networks $\hat{a}\left(x,w_{a}\right)$ and $\hat{b}\left(x,w_{b}\right)$, respectively, expressed as

(32) $${\hat{a}}^{\left(k\right)}={\left[\;{\left({\hat{a}}_{1}^{\left(k\right)}\right)}^{T}\;\;{\left({\hat{a}}_{2}^{\left(k\right)}\right)}^{T}\;\cdots \;{\left({\hat{a}}_{i}^{\left(k\right)}\right)}^{T}\;\cdots \;{\left({\hat{a}}_{M}^{\left(k\right)}\right)}^{T}\;\right]}^{T}$$

and

(33) $${\hat{b}}^{\left(k\right)}={\left[\;{\left({\hat{b}}_{1}^{\left(k\right)}\right)}^{T}\;\;{\left({\hat{b}}_{2}^{\left(k\right)}\right)}^{T}\;\cdots \;{\left({\hat{b}}_{i}^{\left(k\right)}\right)}^{T}\;\cdots \;{\left({\hat{b}}_{M}^{\left(k\right)}\right)}^{T}\;\right]}^{T}$$

where i=1,2,...,M.

In the fifth stage, we train the proposed neuro-TF model. First, a preliminary training process [22] is performed for the two neural networks $\hat{a}\left(x,w_{a}\right)$ and $\hat{b}\left(x,w_{b}\right)$ using the obtained training data $\left(x_{k},{\hat{a}}^{\left(k\right)}\right)$ and $\left(x_{k},{\hat{b}}^{\left(k\right)}\right)$, respectively. The two neural networks are trained separately to learn the relationships between the geometrical variables x and the coefficients (i.e., $\hat{a}$ and $\hat{b}$). After the preliminary training, we combine the trained neural networks with the transfer functions to obtain the overall proposed neuro-TF model. Even though the neural networks are trained well, the training and testing errors of the overall proposed model may still not satisfy the accuracy criteria. To make the overall proposed model satisfy the accuracy criteria, we perform a model refinement training process [22] to refine the overall proposed model. The training data for the proposed overall model are ($x_{k}$, $d_{k}$), where $k\in T_{r}$. The objective of the training is to minimize the training error $E_{Tr}$ of the overall model by optimizing the neural network weights $w_{a}$ and $w_{b}$. The training error function $E_{Tr}$ is formulated as

(34) $$E_{Tr}\left(w_{a},w_{b}\right)=\;\frac{1}{2n_{s}}\;\sum_{k\in T_{r}}\sum_{\lambda \in \Omega }{∥\sum_{i=1}^{M}y_{i}\left(w_{a},w_{b},x_{k},s_{\lambda }\right)-d_{k,\lambda }∥}^{2}$$

where $n_{s}$ denotes the total number of training samples; Ω denotes the index set of frequency samples; *M* represents the number of the sub-neuro-TF models; and $y_{i}$ represents the output of the *i*th sub-neuro-TF model, which is also a function of $x_{k}$, $w_{a}$ and $w_{b}$, and $s_{\lambda }$.

We use the training data ($x_{k}$, $d_{k}$) to verify the quality of trained overall model. If the training error $E_{Tr}$ is lower than a user-defined threshold $E_{t}$, the whole training process is terminated and the model is ready for testing. Otherwise, the two neural networks are under-learned. In that case, we should increase the number of hidden neurons for the two neural networks and repeat the two training processes.

After the model is trained, we test the quality of the model using independent testing data which are never used in training. We define $E_{Ts}$ to be the testing error. If the testing error $E_{Ts}$ is also lower than the threshold $E_{t}$, the model development is finished and the current value of *M* is regarded as the final *M*. Otherwise, we increase the number *M* of sub-neuro-TF models by one (i.e., M=M+1) and repeat the parameter extraction and training processes from the third stage to the fifth stage (25)–(34) of the proposed decomposition technique.

Here we summarize the proposed decomposition technique in a stepwise algorithm as follows.

*Step* *1:* Select the effective poles and residues ($p^{\left(k\right)}$ and $r^{\left(k\right)}$) by (12)–(18).
*Step* *2:* Obtain the deviation vector σ by (19)–(24).
*Step* *3:* Initialize the number *M* of the sub-neuro-TF models to two.
*Step* *4:* Obtain the effective order $N_{i}$ by (25) and subset $I_{i}$ by (26)–(29) with the current value of *M* for the *i*th sub-neuro-TF model.
*Step* *5:* Obtain the pole and residue data ${\hat{p}}_{i}^{\left(k\right)}$ and ${\hat{r}}_{i}^{\left(k\right)}$ by (30)–(31) for the *i*th sub-neuro-TF model. Convert data ${\hat{p}}_{i}^{\left(k\right)}$ and ${\hat{r}}_{i}^{\left(k\right)}$ into data ${\hat{a}}_{i}^{\left(k\right)}$ and ${\hat{b}}_{i}^{\left(k\right)}$. Obtain the training data $\left(x_{k},{\hat{a}}^{\left(k\right)}\right)$ and $\left(x_{k},{\hat{b}}^{\left(k\right)}\right)$ by (32)–(33) for the two neural networks $\hat{a}\left(x,w_{a}\right)$ and $\hat{b}\left(x,w_{b}\right)$.
*Step* *6:* Perform the preliminary training of the two neural networks and refinement training of the overall model by (34).
*Step* *7:* Use the training data ($x_{k}$, $d_{k}$) to verify the trained overall model. If the training error $E_{Tr}$ is lower than a user-defined threshold $E_{t}$, go to *Step 8*. Otherwise, increase the number of hidden neurons and go to *Step 6*.
*Step* *8:* Use the testing data to verify the overall model. If the testing error $E_{Ts}$ is lower than the user-defined threshold $E_{t}$, go to *Step 9*. Otherwise, increase the number *M* of sub-neuro-TF models by one (i.e., M=M+1) and go to *Step 4*.
*Step* *9:* Stop the modeling process.

**Figure 2:**
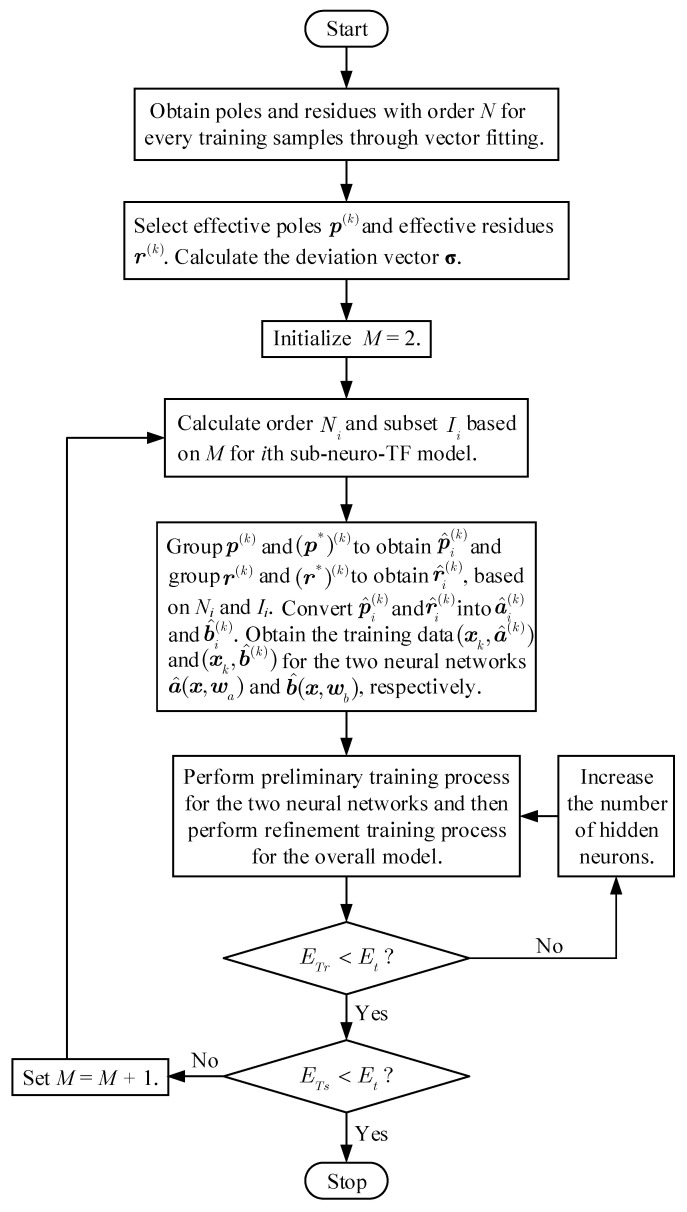
The flow diagram of the overall model development process using the proposed decomposition technique.

Figure 2 shows a flow diagram of the development process for the overall neuro-TF model using the proposed decomposition technique.

## 4. Application Examples

### 4.1. Three-Order Waveguide Filter Modeling

**Figure 3:**
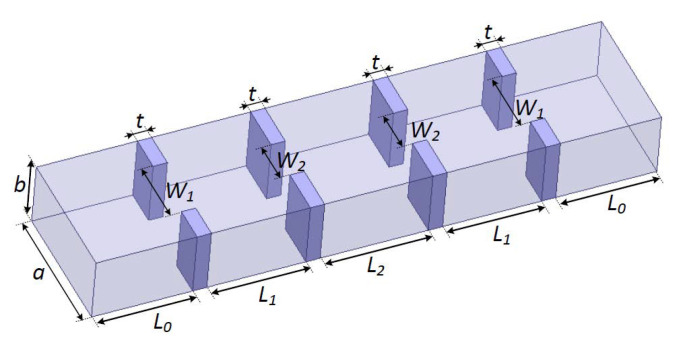
Geometrical variables and 3D configuration of the three-order waveguide filter example.

A three-order waveguide filter [27] is used to illustrate the rational-based neuro-TF method using the proposed decomposition technique. As can be seen in Figure 3, *a* = 19.05 mm, *b* = 9.525 mm, and *t* = 2.0 mm and the geometrical variables are $x={\left[L_{1}\;L_{2}\;W_{1}\;W_{2}\right]}^{T}$ for the three-order waveguide filter. Four geometrical variables, i.e., $x={\left[L_{1}\;L_{2}\;W_{1}\;W_{2}\right]}^{T}$, are used as model inputs and the real and imaginary parts of $S_{11}$, i.e., $y=[RS_{11}$$IS_{11}{]}^{T}$, are used as model outputs.

Full-wave EM simulations are performed using the ANSYS HFSS EM simulator to generate training and test samples for parametric modeling. Both training samples and test samples are generated using the design of experiments (DOE) [28] sampling method. As shown in Table 2, the proposed parametric modeling technique is performed for three cases according to different geometrical parameter ranges. Case 1 is with a narrower range, Case 2 is with an increased range, and Case 3 is with a wider range. In all cases, we set the total order *N* of the overall transfer function to 10 for every sample. Subsequently, $N_{eff}$ equals five for the three cases. In Case 1 and Case 2, the frequency range is from 11.65 GHz to 12.35 GHz and the number of frequency samples per geometrical sample equals 71. In Case 3, the frequency range is from 11.0 GHz to 13.0 GHz and the number of frequency samples per geometrical sample equals 201. We use seven levels of DOE for all cases to generate the training and test samples. The numbers for training samples and testing samples are both 49. Table 2 lists the specific ranges of training samples and test samples for the three cases.

We scale and shift the frequency range and set the total order *N* to ten (an even number) during the vector fitting process, ensuring that the obtained poles and residues are in complex values.

Since the initial value of the number *M* of sub-neuro-TF models is set to two, the proposed model is initially trained with *M* = 2. Using the proposed decomposition technique with *M* = 2, the effective orders are determined as $N_{1}$ = 3 and $N_{2}$ = 2 for all the three cases and the subsets are determined as $I_{1}$ = {2,4,5} and $I_{2}$ = {1,3} for Case 1 and Case 2 and $I_{1}$ = {2,3,4} and $I_{2}$ = {1,5} for Case 3. The proposed neuro-TF model with *M*=2 has a frequency variable for each sub-neuro-TF model, four input geometrical variables, and two outputs. The overall transfer function consists of two sub-transfer functions. The high effective order $N_{eff}$ of 5 is decomposed into lower effective orders $N_{1}$ of 3 and $N_{2}$ of 2.

**Figure 4:**
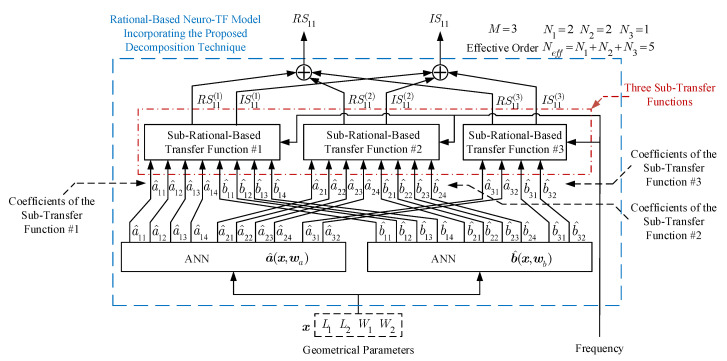
Structure for the proposed rational-based neuro-TF model with M=3 of the three-order waveguide filter example.

The NeuroModelerPlus software is used for training of the proposed model. Suitable numbers of hidden neurons are used to achieve a good learning of the proposed neuro-TF model. The user-defined threshold $E_{t}$ for the three-order waveguide filter example is set to 1%. Three parametric models are developed for the three cases using the proposed technique. The average training errors of the trained models with M=2 are 0.239%, 0.467%, and 1.490% for Case 1, Case 2, and Case 3, respectively. The average testing errors of the trained models with M=2 are 0.267%, 0.496%, and 1.840% for Case 1, Case 2, and Case 3, respectively. On the one hand, since the training and testing errors for both Case 1 and Case 2 are lower than $E_{t}$, the two trained models with M=2 are the final models for both Case 1 and Case 2. On the other hand, since the testing error for Case 3 is higher than $E_{t}$, we increased *M* to three and redeveloped the model with M=3 for Case 3. Figure 4 illustrates the model structure with *M* = 3 for the three-order waveguide filter example. As illustrated in Figure 4, the overall transfer function consists of three sub-transfer functions. The high effective order $N_{eff}$ of 5 is decomposed into lower effective orders $N_{1}$ of 2, $N_{2}$ of 2, and $N_{3}$ of 1. The subsets are determined as $I_{1}$ = {2,4}, $I_{2}$ = {3,5}, and $I_{3}$ = {1} for Case 3. The average training and testing errors of the trained models with M=3 are 0.746% and 0.962%, which are lower than $E_{t}$. Therefore, the trained model with M=3 is the final model for Case 3. The number of different parameter extractions in the proposed technique depends on both *M* and the number $n_{s}$ of total training samples. Specifically, the number of different parameter extractions equals $n_{s}$ multiplied by (*M*-1). In this example, we performed 49 different parameter extractions for Cases 1 and 2, and 98 different parameter extractions for Case 3.

For comparison purpose, we apply the existing modeling approach using rational-based neuro-TF [25] to the three separate cases of this example.

Comparisons of number *M* of sub-neuro-TF models, effective order $N_{i}$ for *i*th sub-neuro-TF model, hidden neuron numbers, and average errors for training and testing between different rational-based neuro-TF modeling methods and EM data are shown in Table 3.

In Case 1, since the geometrical variations are small, the relatively low nonlinear relationship between the geometrical variables and the coefficients of the transfer function can be easily represented by the neural networks with less hidden neurons of both the existing model and the proposed model in the preliminary training. With the high training accuracy of the neural networks of both models, both models can have high training accuracy in the refinement training. For these reasons, both methods in this case can obtain good training and testing accuracy.

In Case 2, since the geometrical variables vary within an increased range, the relationship between the geometrical variables and the coefficients of the transfer function becomes more nonlinear than that in Case 1. In this case, the sensitivities of the overall model response with respect to the coefficients of the transfer function have higher effects on the accuracy of the overall model. Since the orders and the sensitivities for the proposed model are much lower than those for the existing model, the proposed model can obtain more accurate training and testing results than the existing method.

In Case 3, the geometrical variables vary within a wider range, the relationship between the geometrical variables and the coefficients of the transfer function becomes highly nonlinear. In this case, the sensitivities of the overall model response with respect to the coefficients of the transfer function have large effects on the accuracy of the overall model. The proposed model with M=2 have smaller training and testing errors than the existing model but still can not satisfy the model accuracy criteria. Therefore, we increased the *M* to be 3. With M=3, the proposed model can satisfy the model accuracy criteria. It can be concluded from the above three cases that the neuro-TF model using the proposed decomposition technique has much lower sensitivities of the overall model response with respect to the coefficients of the transfer function, improving the modeling accuracy over the existing method.

**Figure 5:**
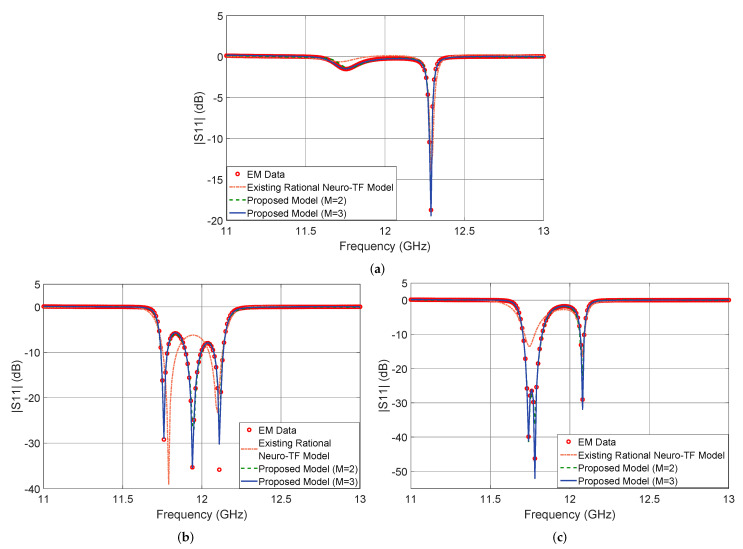
Comparison of magnitude in decibels of $S_{11}$ different modeling approaches and EM data: (**a**) test sample x=[14.37 14.57 9.25 6.17${]}^{T}$ (mm), (**b**) test sample x=[14.17 15.20 8.75 6.25${]}^{T}$ (mm), and (**c**) test sample x=[14.56 15.20 8.63 6.17${]}^{T}$ (mm) for the three-order waveguide filter example.

**Figure 6:**
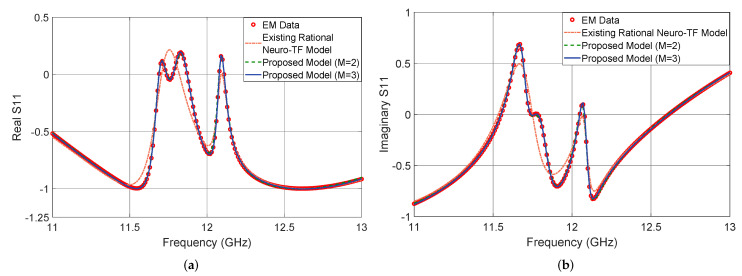
Comparison of Re(S11) and Im(S11) for different modeling approaches and EM data at test sample x=[14.56 15.20 8.63 6.17${]}^{T}$ (mm) for the three-order waveguide filter example. (**a**) Re(S11) and (**b**) Im(S11).

Figure 5 shows the output $|S_{11}|$ in dB of the proposed neuro-TF model for three different test geometrical samples in Case 3 of the three-order waveguide filter example. We compare the model response using the proposed technique with M=3, using the proposed technique with M=2, using the existing neuro-TF method, and EM data. We also provide comparisons of the real and imaginary parts of $S_{11}$, as shown in Figure 6. The geometrical variables for the three test samples are x=[14.37 14.57 9.25 6.17${]}^{T}$ (mm), x=[14.17 15.20 8.75 6.25${]}^{T}$ (mm), and x=[14.56 15.20 8.63 6.17${]}^{T}$ (mm). These three test samples belong to Case 3.

It is observed from Figure 5 and Figure 6 that the neuro-TF model using the proposed decomposition method can achieve better model accuracy for different geometrical samples which are never used in training.

### 4.2. Four-Order Bandpass Filter Modeling

**Figure 7:**
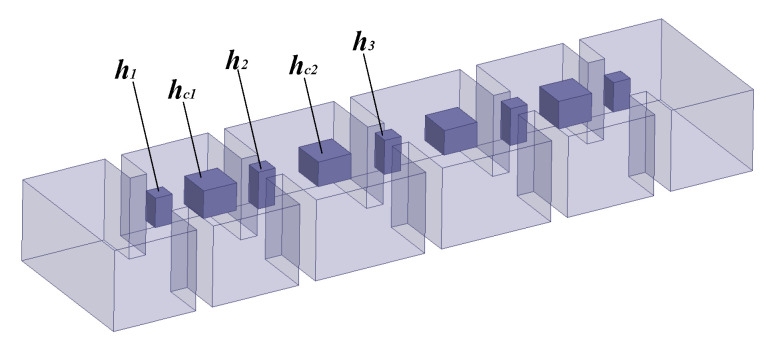
Geometrical variables and 3D configuration of the four-order bandpass filter example.

A four-order bandpass filter [29] is used to illustrate the rational-based neuro-TF method using the proposed decomposition technique. As can be seen in Figure 7, the geometrical variables are $x={\left[h_{1}\;h_{2}\;h_{3}\;h_{c1}\;h_{c2}\right]}^{T}$ for the four-order bandpass filter, where $h_{1}$, $h_{2}$, and $h_{3}$ denote the heights of the posts between the coupling windows, and $h_{c1}$ and $h_{c2}$ denote the heights of the posts in the resonant cavities. These five geometrical variables are used as model inputs and the real and imaginary parts of $S_{11}$ are used as model outputs.

Similar to the first example, ANSYS HFSS EM simulator and DOE sampling method are used for generation of training and test samples. As shown in Table 4, the proposed parametric modeling technique is performed for two different cases according to different geometrical parameter ranges. Case 1 is with a narrower parameter range and Case 2 is with a wider parameter range. In both cases, we set the total order *N* of the overall transfer function to 12 for every sample. Subsequently, $N_{eff}$ equals six for the two cases. In both cases, the frequency range is from 10.50 GHz to 11.50 GHz and the number of frequency samples per geometrical sample equals 101. We use nine levels of DOE to generate the training samples and eight levels of DOE to generate the test samples for both cases. The numbers for the training samples and the testing samples are 81 and 64, respectively. Table 4 lists the specific ranges of training samples and test samples for the two cases.

Similar to the first example, the proposed model is initially trained with M=2. With M=2, the effective orders are determined as $N_{1}=3$ and $N_{2}=3$ for both cases and the subsets are determined as $I_{1}=\left\{2,4,5\right\}$ and $I_{2}=\left\{1,3,6\right\}$ for both cases. The proposed neuro-TF model with M=2 has a frequency variable for each sub-neuro-TF model, five input geometrical variables, and two outputs. The overall transfer function consists of two sub-transfer functions. The high effective order $N_{eff}$ of 6 is decomposed into lower effective orders $N_{1}$ of 3 and $N_{2}$ of 3.

**Figure 8:**
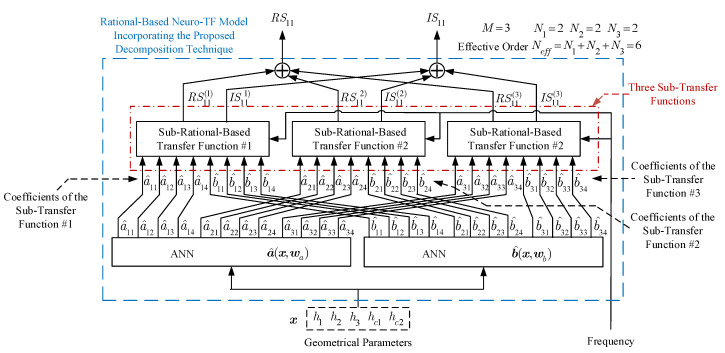
Structure for the proposed rational-based neuro-TF model with M=3 of the four-order bandpass filter example.

The NeuroModelerPlus software is used for training of the proposed model. The user-defined threshold $E_{t}$ for the four-order bandpass filter example is set to 2%. Two parametric models are developed for the two cases using the proposed technique. The average training errors of the trained models with M=2 are 1.487% and 2.252% for Case 1 and Case 2, respectively. The average testing errors of the trained models with M=2 are 1.672% and 4.397% for Case 1 and Case 2, respectively. On the one hand, since the training and testing errors for Case 1 are lower than $E_{t}$, the trained model with M=2 is the final model for Case 1. On the other hand, since the testing error for Case 2 is higher than $E_{t}$, we increased *M* to three and redeveloped the model with M=3 for Case 2. Figure 8 illustrates the model structure with *M* = 3 for the four-order bandpass filter example. As illustrated in Figure 8, the overall transfer function consists of three sub-transfer functions. The high effective order $N_{eff}$ of 6 is decomposed into lower effective orders $N_{1}$ of 2, $N_{2}$ of 2, and $N_{3}$ of 2. The subsets are determined as $I_{1}=\left\{2,5\right\}$, $I_{2}=\left\{3,4\right\}$, and $I_{3}=\left\{1,6\right\}$ for Case 2. The average training and testing errors of the trained model with M=3 are 1.624% and 1.982%, respectively, which are lower than $E_{t}$. Therefore, the trained model with M=3 is the final model for Case 2. In this example, we performed 162 different parameter extractions for each case.

For comparison purpose, we apply the existing modeling approach using rational-based neuro-TF to the two separate cases of this example. Comparisons of number *M* of sub-neuro-TF models, effective order $N_{i}$ for *i*th sub-neuro-TF model, hidden neuron numbers, and average errors for training and testing between different rational-based neuro-TF modeling methods and EM data are shown in Table 5.

In Case 1, since the geometrical variations are relatively small, the relationship between the geometrical variables and the coefficients of the transfer function is moderately nonlinear. In this case, the sensitivities of the overall model response with respect to the coefficients of the transfer function have moderate effects on the accuracy of the overall model. Since the orders and the sensitivities for the proposed model are much lower than those for the existing model, the proposed model can obtain more accurate training and testing results than the existing method.

In Case 2, the geometrical variables vary within a wider range, the relationship between the geometrical variables and the coefficients of the transfer function becomes highly nonlinear. In this case, the sensitivities of the overall model response with respect to the coefficients of the transfer function have large effects on the accuracy of the overall model. The proposed model with M=2 have smaller training and testing errors than the existing model but still can not satisfy the model accuracy criteria. Therefore, we increased the *M* to be 3. With M=3, the proposed model can satisfy the model accuracy criteria. It can be again concluded from the above two cases that the neuro-TF model using the proposed decomposition technique has much lower sensitivities of the overall model response with respect to the coefficients of the transfer function, improving the modeling accuracy over the existing method.

**Figure 9:**
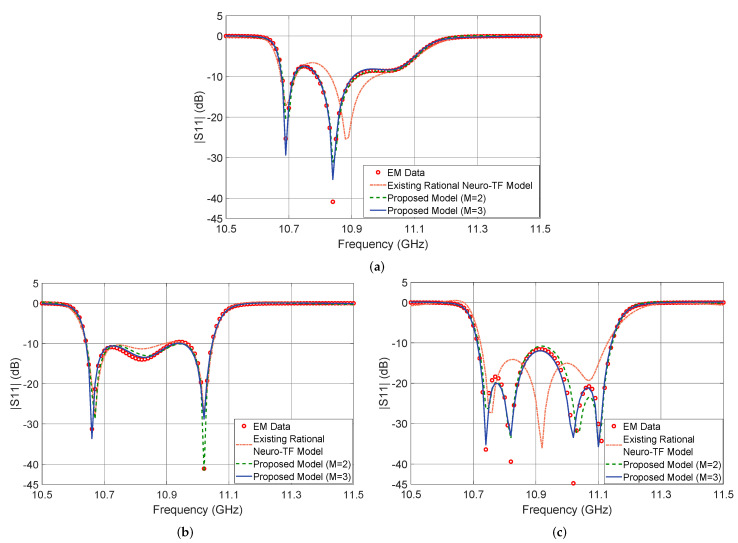
Comparison of magnitude in decibels of $S_{11}$ for different modeling approaches and EM data: (**a**) test sample x=[3.52 4.46 3.96 3.28 3.07${]}^{T}$ (mm), (**b**) test sample, x=[3.60 4.22 4.20 3.40 3.10${]}^{T}$ (mm), and (**c**) test sample x=[3.36 4.38 4.00 3.36 3.02${]}^{T}$ (mm) for the four-order bandpass filter example.

**Figure 10:**
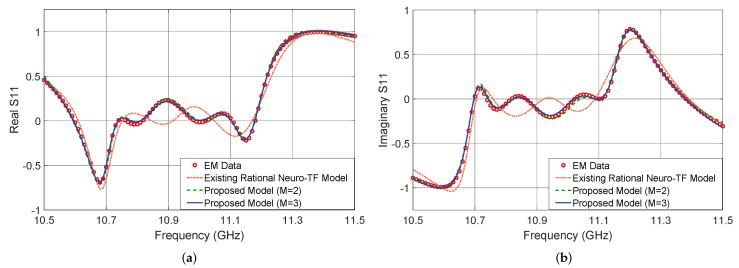
Comparison of Re(S11) and Im(S11) for different modeling approaches and EM data at test sample x=[3.36 4.38 4.00 3.36 3.02${]}^{T}$ (mm) for the four-order bandpass filter example. (**a**) Re(S11) and (**b**) Im(S11).

Figure 9 shows the output $|S_{11}|$ in dB of the proposed neuro-TF model for three different test geometrical samples in Case 2 of the four-order bandpass filter example. We compare the model response using the proposed technique with M=3, using the proposed technique with M=2, using the existing neuro-TF method, and EM data. We also provide comparisons of the real and imaginary parts of $S_{11}$, as shown in Figure 10. The geometrical variables for the three test samples are x=[3.52 4.46 3.96 3.28 3.07${]}^{T}$ (mm), x=[3.60 4.22 4.20 3.40 3.10${]}^{T}$ (mm), and x=[3.36 4.38 4.00 3.36 3.02${]}^{T}$ (mm).

It is observed from Figure 9 and Figure 10 that the neuro-TF model using the proposed decomposition method can achieve better model accuracy for different geometrical samples which are never used in training.

## 5. Conclusions

This paper has proposed a novel decomposition technique for rational-based neuro-TF modeling method. The proposed technique has decomposed the single neuro-TF model with high order into multiple sub-neuro-TF models with the much lower order and reformulated the overall neuro-TF model as the combination of the sub-neuro-TF models. New formulations have been derived for the determination of the number of sub-neuro-TF models and the order of each sub-neuro-TF model. Since the high order of the overall neuro-TF model has been decomposed into multiple lower orders of the sub-neuro-TF models, the proposed technique has decreased the sensitivities of the overall model response with respect to the coefficients of the transfer function. By decreasing the sensitivities using the proposed decomposition technique, the accuracy of the overall neuro-TF model has been improved. In this paper, the proposed decomposition technique has been applied to two microwave filter applications. The potential use of the proposed technique for other EM applications, such as diplexers and antennas, could be an interesting future extension of this work.

## Figures and Tables

**Table 1:** Comparison of the sensitivity of rational-based neuro-transfer function (neuro-TF) model response with respect to the coefficients with/without decomposition.

	Coeff.	Transfer Function	Sensitivity of Transfer Function Response w.r.t the Coeff.
Existing Rational-Based Neuro-TF model (without Decom-position)	$a_{j}$	$y=\frac{\sum_{j=1}^{N}a_{j}s^{j-1}}{1+\sum_{j=1}^{N}b_{j}s^{j}}$	$\frac{\partial y}{\partial a_{j}}=\frac{s^{j-1}}{1+\sum_{j=1}^{N}b_{j}s^{j}}=\frac{s^{j-1}}{\prod_{j=1}^{N_{eff}}\left(s-p_{j}\right)\cdot \prod_{j=1}^{N_{eff}}\left(s-p_{j}^{*}\right)}$
	$b_{j}$		$\frac{\partial y}{\partial b_{j}}=\frac{-s^{j}\sum_{j=1}^{N}a_{j}s^{j-1}}{{\left(1+\sum_{j=1}^{N}b_{j}s^{j}\right)}^{2}}=\frac{-y\cdot s^{j}}{\prod_{j=1}^{N_{eff}}\left(s-p_{j}\right)\cdot \prod_{j=1}^{N_{eff}}\left(s-p_{j}^{*}\right)}$
Proposed Rational-Based Neuro-TF model (with Decom-position)	${\hat{a}}_{ij}$	$y=\sum_{i=1}^{M}y_{i}=\sum_{i=1}^{M}\frac{\sum_{j=1}^{2N_{i}}{\hat{a}}_{ij}s^{j-1}}{1+\sum_{j=1}^{2N_{i}}{\hat{b}}_{ij}s^{j}}$	$\frac{\partial y}{\partial {\hat{a}}_{ij}}=\frac{s^{j-1}}{1+\sum_{j=1}^{2N_{i}}{\hat{b}}_{ij}s^{j}}=\frac{s^{j-1}}{\prod_{j=1}^{N_{i}}\left(s-p_{n(i,j)}\right)\cdot \prod_{j=1}^{N_{i}}\left(s-p_{n(i,j)}^{*}\right)}$
	${\hat{b}}_{ij}$		$\frac{\partial y}{\partial {\hat{b}}_{ij}}=\frac{-s^{j}\sum_{j=1}^{2N_{i}}{\hat{a}}_{ij}s^{j-1}}{{\left(1+\sum_{j=1}^{2N_{i}}{\hat{b}}_{ij}s^{j}\right)}^{2}}=\frac{-y_{i}\cdot s^{j}}{\prod_{j=1}^{N_{i}}\left(s-p_{n(i,j)}\right)\cdot \prod_{j=1}^{N_{i}}\left(s-p_{n(i,j)}^{*}\right)}$

**Table 2:** Definition of training and test samples for the three-order waveguide filter example.

Geometrical Parameters (mm)		Training Samples (49 Samples)			Test Samples (49 Samples)		
		**Min**	**Max**	**Steps**	**Min**	**Max**	**Steps**
Case 1 (Narrower Range)	$L_{1}$	13.84	14.12	0.05	13.86	14.10	0.04
	$L_{2}$	15.05	15.35	0.05	15.07	15.33	0.04
	$W_{1}$	8.91	9.09	0.03	8.93	9.08	0.03
	$W_{2}$	5.94	6.06	0.02	5.95	6.05	0.02
Case 2 (Increased Range)	$L_{1}$	13.70	14.26	0.09	13.75	14.21	0.08
	$L_{2}$	14.90	15.50	0.10	14.95	15.45	0.08
	$W_{1}$	8.82	9.18	0.06	8.85	9.15	0.05
	$W_{2}$	5.88	6.12	0.04	5.90	6.10	0.03
Case 3 (Wider Range)	$L_{1}$	13.28	14.68	0.23	13.40	14.56	0.19
	$L_{2}$	14.44	15.96	0.25	14.57	15.83	0.21
	$W_{1}$	8.55	9.45	0.15	8.63	9.38	0.13
	$W_{2}$	5.70	6.30	0.10	5.75	6.25	0.08

**Table 3:** Comparisons of different rational-based neuro-TF modeling approaches for the three-order waveguide filter example.

Modeling Methods		No. of Sub-Models	$N_{\mathrm{eff}}$ or $N_{i}$	No. of Hidden Neurons		Average Training Error	Average Testing Error
Case 1 (Narrower Range)	Existing Rational Neuro-TF Method	1	5	NN for Numerator	10	0.630 %	0.718 %
				NN for Denominator	10		
	Proposed Rational Neuro-TF Method	2	3 2	NN for Numerator	10	0.239 %	0.267 %
				NN for Denominator	10		
Case 2 (Increased Range)	Existing Rational Neuro-TF Method	1	5	NN for Numerator	10	1.953%	2.017%
				NN for Denominator	10		
	Proposed Rational Neuro-TF Method	2	3 2	NN for Numerator	10	0.467%	0.496%
				NN for Denominator	10		
Case 3 (Wider Range)	Existing Rational Neuro-TF Method	1	5	NN for Numerator	10	5.073%	6.604%
				NN for Denominator	10		
		1	5	NN for Numerator	40	3.222%	50.69%
				NN for Denominator	40		
	Proposed Rational Neuro-TF Method	2	3 2	NN for Numerator	10	1.490%	1.840%
				NN for Denominator	10		
		3	2 2 1	NN for Numerator	10	0.746%	0.962%
				NN for Denominator	10		

**Table 4:** Definition of training and test samples for the four-order bandpass filter example.

Geometrical Parameters (mm)		Training Samples (81 Samples)			Test Samples (64 Samples)		
		**Min**	**Max**	**Steps**	**Min**	**Max**	**Steps**
Case 1 (Narrower Range)	$h_{1}$	3.4	3.56	0.02	3.41	3.55	0.02
	$h_{2}$	4.3	4.46	0.02	4.31	4.45	0.02
	$h_{3}$	4.0	4.16	0.02	4.01	4.15	0.02
	$h_{c1}$	3.2	3.36	0.02	3.21	3.35	0.02
	$h_{c2}$	2.9	3.06	0.02	2.91	3.05	0.02
Case 2 (Wider Range)	$h_{1}$	3.3	3.62	0.04	3.32	3.6	0.04
	$h_{2}$	4.2	4.52	0.04	4.22	4.5	0.04
	$h_{3}$	3.9	4.22	0.04	3.92	4.2	0.04
	$h_{c1}$	3.1	3.42	0.04	3.12	3.4	0.04
	$h_{c2}$	2.8	3.12	0.04	2.82	3.1	0.04

**Table 5:** Comparisons of different rational-based neuro-TF modeling approaches for the four-order bandpass filter example.

Modeling Methods		No. of Sub-Models	$N_{\mathrm{eff}}$ or $N_{i}$	No. of Hidden Neurons		Average Training Error	Average Testing Error
Case 1 (Narrower Range)	Existing Rational Neuro-TF Method	1	6	NN for Numerator	10	4.448%	4.674%
				NN for Denominator	10		
		1	6	NN for Numerator	40	2.562%	9.121%
				NN for Denominator	40		
	Proposed Rational Neuro-TF Method	2	3 3	NN for Numerator	10	1.487%	1.672%
				NN for Denominator	10		
		3	2 2 2	NN for Numerator	10	0.876%	1.015%
				NN for Denominator	10		
Case 2 (Wider Range)	Existing Rational Neuro-TF Method	1	6	NN for Numerator	10	6.809%	8.382%
				NN for Denominator	10		
		1	6	NN for Numerator	40	4.791%	32.23%
				NN for Denominator	40		
	Proposed Rational Neuro-TF Method	2	3 3	NN for Numerator	10	2.252%	4.397%
				NN for Denominator	10		
		2	3 3	NN for Numerator	40	1.877%	16.21%
				NN for Denominator	40		
		3	2 2 2	NN for Numerator	10	1.624%	1.982%
				NN for Denominator	10		
